# Vitamin C may reduce the duration of mechanical ventilation in critically ill patients: a meta-regression analysis

**DOI:** 10.1186/s40560-020-0432-y

**Published:** 2020-02-07

**Authors:** Harri Hemilä, Elizabeth Chalker

**Affiliations:** 1 Department of Public Health, University of Helsinki, POB 41, FI-00014 Helsinki, Finland; 2 University of Sydney, Sydney, Australia

**Keywords:** Antioxidants, Burns, Artificial respiration, Cardiac surgical procedures, Critical care, Dietary supplements, Oxidative stress, Meta-analysis, Sepsis, Systematic review

## Abstract

**Background:**

Our recent meta-analysis indicated that vitamin C may shorten the length of ICU stay and the duration of mechanical ventilation. Here we analyze modification of the vitamin C effect on ventilation time, by the control group ventilation time (which we used as a proxy for severity of disease in the patients of each trial).

**Methods:**

We searched MEDLINE, Scopus, and the Cochrane Central Register of Controlled Trials and reference lists of relevant publications. We included controlled trials in which the administration of vitamin C was the only difference between the study groups. We did not limit our search to randomized trials and did not require placebo control. We included all doses and all durations of vitamin C administration. One author extracted study characteristics and outcomes from the trial reports and entered the data in a spreadsheet. Both authors checked the data entered against the original reports. We used meta-regression to examine whether the vitamin C effect on ventilation time depends on the duration of ventilation in the control group.

**Results:**

We identified nine potentially eligible trials, eight of which were included in the meta-analysis. We pooled the results of the eight trials, including 685 patients in total, and found that vitamin C shortened the length of mechanical ventilation on average by 14% (*P* = 0.00001). However, there was significant heterogeneity in the effect of vitamin C between the trials. Heterogeneity was fully explained by the ventilation time in the untreated control group. Vitamin C was most beneficial for patients with the longest ventilation, corresponding to the most severely ill patients. In five trials including 471 patients requiring ventilation for over 10 h, a dosage of 1–6 g/day of vitamin C shortened ventilation time on average by 25% (*P* < 0.0001).

**Conclusions:**

We found strong evidence that vitamin C shortens the duration of mechanical ventilation, but the magnitude of the effect seems to depend on the duration of ventilation in the untreated control group. The level of baseline illness severity should be considered in further research. Different doses should be compared directly in future trials.

## Background

In controlled trials, vitamin C has improved endothelial function, lowered blood pressure, increased left ventricular ejection fraction, decreased the incidence of atrial fibrillation, decreased bronchoconstriction, prevented pain, shortened the duration of colds, and decreased the incidence of colds in physically stressed people, and it may also have beneficial effects against pneumonia, see reference [1].

The average person, in good health, maintains normal plasma vitamin C levels with a daily intake of about 0.1 g/day. However, much higher doses, in the order of grams per day, are needed for critically ill patients to reach normal plasma vitamin C levels [2–5]. Without supplementation, plasma vitamin C levels are particularly low in critically ill patients [6–10], indicating that the body may have a greater need for vitamin C when under severe stress such as illness requiring intensive care. It seems evident that there are gradual changes in vitamin C metabolism according to the severity of disease, in that the sicker a patient is, the greater the consumption of vitamin C. This further suggests that the sicker a patient is, the more they are likely to benefit from additional vitamin C.

Given this background, we previously examined whether vitamin C administration has an effect on practical outcomes such as the length of ICU stay, without looking at specific medical conditions. From the results of 12 trials with 1766 patients, we calculated that vitamin C reduced the length of ICU stay on average by 7.8% (*P* < 0.001) [1].

We also found that in trials in which the control groups were ventilated for 24 h or more, vitamin C shortened the duration of mechanical ventilation by 18% (*P* = 0.001) [1]. However, vitamin C did not have an effect on the duration of mechanical ventilation in trials in which control groups were ventilated for less than 24 h, i.e., trials in patients with less severe illness.

In this study, we hypothesize that there is a continuous relationship between disease severity and the beneficial effect of vitamin C administration. We used meta-regression to analyze the gradual relationship between the effect of vitamin C in the treatment group and the duration of mechanical ventilation in untreated patients of the control group, which we used as a proxy for the severity of the disease.

## Methods

We included controlled trials that compared the length of mechanical ventilation between vitamin C and control groups. We included trials in which the administration of vitamin C was the only difference between the study groups; trials that administered other therapies as well as vitamin C were included only if the other therapies were the same for both trial groups. We did not limit our search to randomized trials and did not require placebo control. We included all doses and all durations of vitamin C administration.

**Fig. 1:**
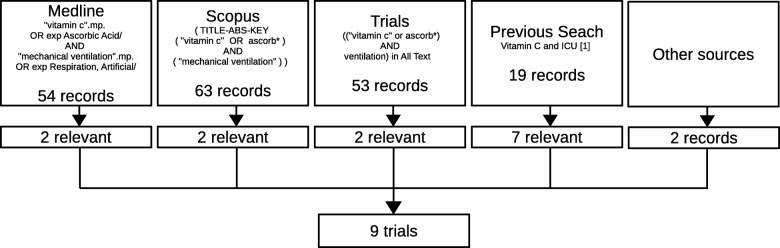
Flow diagram showing the search terms. The searches were carried out on 13 Nov 2019. The searches identified nine trials that we included in our systematic review analysis [11–19] and eight of them were included in our meta-analysis [11–18]

We searched MEDLINE, Scopus, and the Cochrane Register on 13 Nov 2019 with the search phrases described in Fig. 1. We had previously searched the same databases for trials on vitamin C and ICU length of stay on 20 Jan 2019 [1]; from that search, we found trials that were not well indexed and so not identified in our new search specifically for trials on vitamin C and mechanical ventilation. Finally, we perused the reference lists of the selected trials and relevant reviews, from which we discovered two more trials. We identified nine trials satisfying our selection criteria [11–19]. We did not include two further trials that reported “ventilator-free days” since we were unable to convert this to our outcome of interest, “duration of mechanical ventilation” [20, 21].

Tanaka et al. administered vitamin C continuously with a dosage of 66 mg/kg/h for the first 24 h only, with the reported mean weight of patients being 57 kg [17]. Thus, we calculated that the average dose was 90 g/day in the Tanaka trial. Zabet et al. administered 25 mg/kg every 6 h, but the mean weight was not reported [18]. Assuming a mean weight of 60 kg, we estimated that the average dose was 6 g/day in the Zabet trial. We use these estimates in our text.

The outcome in this analysis is the length of mechanical ventilation, which we analyzed on the relative scale. The relative scale is usually more informative than the absolute scale for the analysis of treatment effects on continuous outcomes, in particular, for the analysis of duration data [1, 22–24]. We used the ratio of means (RoM) to estimate the effect of vitamin C, and the Taylor series-based approach to calculate the log(RoM) [22].

We pooled the selected trials with the *metagen* function of the *meta* package of the R statistical software [25–27], using the inverse variance, fixed effect options. For meta-regression, we used the *metareg* function of the *meta* package. We used the *χ*^2^ test and the *I*^2^ statistic to assess statistical heterogeneity among the trials in the meta-analysis [28]. Values of *I*^2^ range from 0% to 100%. A value close to 0% indicates very low-level heterogeneity. A value of more than approximately 50% indicates moderate heterogeneity, and a value over 75% indicates high-level heterogeneity. Our calculations are described in Additional files 1 and 2.

## Results

### Description of the included trials

Nine controlled trials have reported on vitamin C administration and the length of mechanical ventilation (Table 1; see Additional file 1: Table S1 for further details of the trials). The flow diagram of our search is shown in Fig. 1.

**Table 1:** Description of the included trials

Trial [ref.]	*N*	Setting	Vitamin C		Length of mechanical ventilation (hours)				RoM
			Route	Dose (g/day)	Vitamin C		Control		
					Mean	SD	Mean	SD	
Bjordahl et al. [11]	185	Cardiac	po	2	28.8	19.2	33.6	24.0	0.86
Amini et al. [12]	137	Cardiac	po	3	7.3	6.0	6.7	4.3	1.10
Dehghani et al .[13]	100	Cardiac	po	1	13.4	2.0	15.4	14.3	0.87
Habib et al. [14]	100	Sepsis	iv	6	110	50	189	72	0.58
Safaei et al. [15]	58	Cardiac	iv	2	15.1	5.39	22.9	20.46	0.66
Ebade et al. [16]	40	Cardiac	iv	3	2.04	0.35	1.99	0.31	1.02
Tanaka et al. [17]	37	Burns	iv	90*	290	211	511	374	0.57
Zabet et al. [18]	28	Sepsis	iv	6*	36.6	16.1	46.8	10.1	0.78
Sadeghpour et al. [19]**	290	Cardiac	po	1	11.8	3.9	14.1	9.5	0.84

The trials are listed by the number of patients (*N*). The mean age in the trials ranged from 42 to 64 years, with a median of 60 years. The proportion of males varied from 58% to 75%. Five trials were carried out in Iran [12, 13, 15, 18, 19], two in Egypt [14, 16], one in the USA [11], and one in Japan [17]. For detailed descriptions of the trials, see Additional file 1: Table S1. The Amini trial [12] results are modified as described previously [1]

*Estimated vitamin C dose, see the “Methods” section

**Sadeghpour et al. [19] recruited 500 participants but reported the results for just 290 participants [1]. Because of the high dropout rate (42%), we did not include this trial in our calculations, but we overlay the findings in Fig. 4

*iv* intravenous, *po* per oral, *RoM* ratio of means [22]: e.g., for the Bjordahl trial [11], RoM = 0.86, based on 28.8/33.6, and RoM = 0.86 indicates that ventilation time in the vitamin C group was 14% shorter than in the placebo group

The total number of patients was 975, with 810 patients in six cardiac surgery trials, 128 patients in two sepsis trials, and 37 patients in one trial with burns patients (Table 1). Vitamin C was administered orally in four trials and intravenously in five trials. The Tanaka [17] trial administered 90 g/day, whereas the other seven trials administered from 1 to 6 g/day. Safaei et al. [15] and Tanaka et al. [17] administered vitamin C on a single day only, six trials administered for 2–5 days [11–13, 16, 18, 19], and one administered “until ICU discharge” for septic shock patients [14]. There is a 250-fold variation in the average length of mechanical ventilation in the untreated groups from 2 h [16] to 511 h [17], which reflects great variation in the severity of the baseline medical condition (Table 1).

**Fig. 2:**
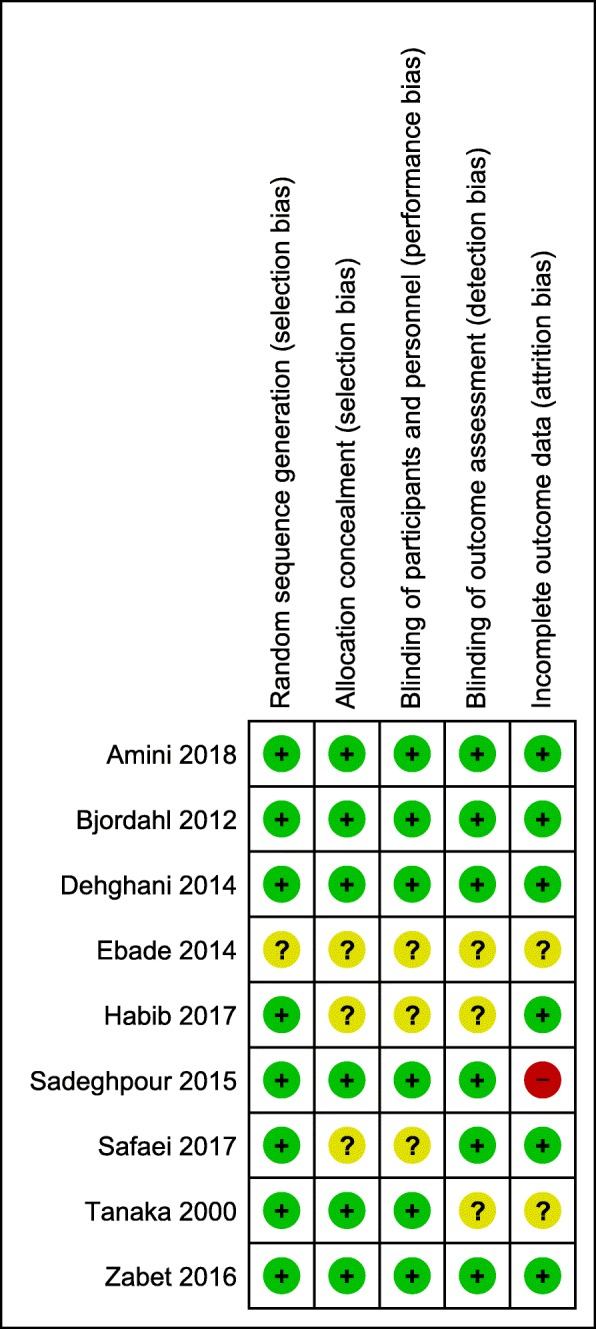
Risk of bias summary. Review authors’ judgments about each risk of bias item for each included trial. A green plus sign (+) indicates that there is no substantial concern for bias in the particular quality item. A question mark (?) indicates that conclusions are unable to be drawn regarding potential bias. A red minus sign (−) indicates that there is an explicit concern regarding bias. In the Sadeghpour trial, the dropout rate was very high (42%), justifying the minus sign [1]. The reference numbers to the trials are shown in Table 1

Six trials were randomized [11–13, 15, 18, 19], two used alternative allocation [14, 17], and one did not describe the allocation method [16]. The reported baseline variables for the treatment groups were balanced in all trials (Additional file 1: Table S1). The risk of bias assessment of the trials is shown in Fig. 2. Four trials used an explicit placebo [11, 16, 18, 19]. The trial by Sadeghpour et al. [19] had a high dropout rate, with 500 participants recruited, but results were reported for just 290 participants [1]. We did not include this trial in our statistical models, but the results are presented separately.

### Results of the included trials

In our standard meta-analyses, we pooled the results of the trials on the relative scale, calculating the ratio of means (RoM) [22]. For example, in the Bjordahl trial [11], the length of mechanical ventilation was 28.8 h in the vitamin C group and 33.6 h in the placebo group, which corresponds to RoM = 0.86 (28.8/33.6) (Table 1). This represents a 14% shorter ventilation time in the vitamin C group.

**Fig. 3:**
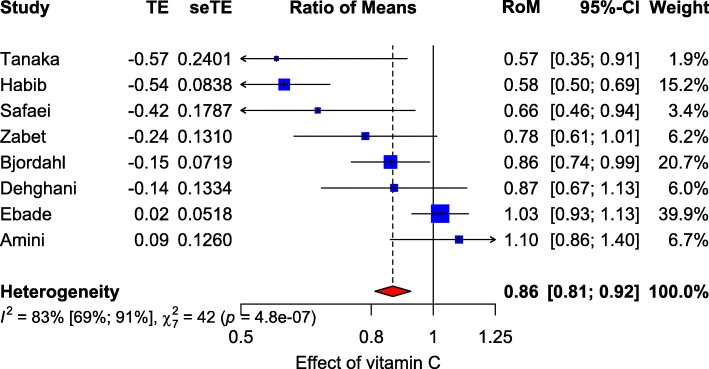
Effect of vitamin C on the duration of ventilation. The horizontal lines indicate the 95% CI for the vitamin C effect and the blue squares in the middle of the horizontal lines indicate the point estimate of the effect in the particular trial. The red diamond shape indicates the pooled effect and its 95% CI. The Sadeghpour trial [19] is not included in the meta-analysis, since the dropout rate was high (42%) [1]. *RoM* ratio of means [22]

Over the eight included trials with 685 patients in total, vitamin C shortened the length of mechanical ventilation on average by 14% (*P* = 10^−5^) (Fig. 3). However, there was highly significant heterogeneity in the vitamin C effect between the trials with *I*^2^ = 83% (*P* = 4.8 × 10^−7^). This indicates that the calculated average effect of 14% is not consistent over all the included trials.

**Fig. 4:**
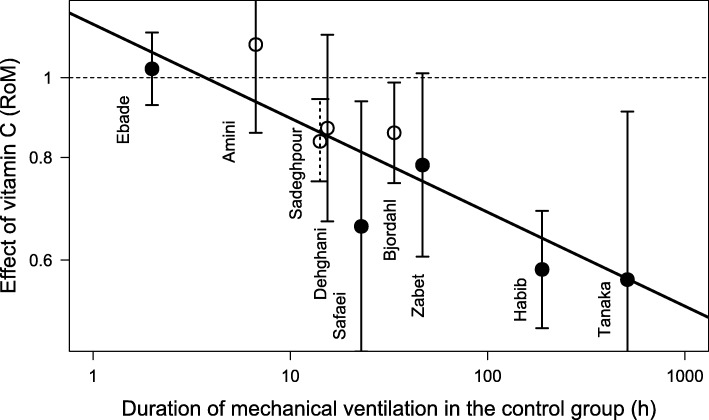
Effect of vitamin C on the duration of ventilation by the duration of ventilation in the control group. The horizontal dashed line indicates the null effect. The diagonal line shows the meta-regression line for the eight trials, with *P* = 10^−7^ for the test that the slope is not null. Vitamin C was administered orally (open circles) or intravenously (filled circles). The regression line follows the formula ln(RoM) = 0.150–0.263 × log_10_(length of ventilation). For example, for a ventilation time of 100 h (log_10_[100] = 2), the formula gives ln(RoM) = − 0.377, and predicts a vitamin C effect of RoM = 0.69, i.e., a 31% shorter ventilation time. The Sadeghpour trial [19] is not included in the statistical model, since the dropout rate was high; however, it is overlaid here for information. For calculations, see Additional file 2. *RoM* ratio of means [22]

In a meta-regression analysis, we found that the heterogeneity between the trials was explained by the length of mechanical ventilation in the untreated control group (Fig. 4). The evidence for modification of the vitamin C effect by the untreated ventilation time was very strong (*P* = 10^−7^). There is little residual heterogeneity around the regression line with *I*^2^ = 12% (*P* = 0.3), which indicates that the meta-regression in Fig. 4 much better captures the findings of the trials compared with the standard meta-analysis shown in Fig. 3. The confidence intervals of all included trials are consistent with the regression line in Fig. 4. The Habib [14] trial contributed considerable weight to the effect of vitamin C in Fig. 4. However, even if both the Habib [14] and Tanaka [17] trials are excluded, there is strong evidence from the six remaining trials that the effect of vitamin C is modified by the ventilation time in the untreated control group (*P* = 0.004) (see Additional file 1).

Figure 4 indicates that no meaningful benefit from vitamin C is expected for patients whose ventilation time is shorter than 10 h. On the other hand, the regression line in Fig. 4 predicts that for patients ventilated for 100 h, vitamin C reduces the ventilation time on average by 31% (RoM = 0.69). Three trials administered vitamin C orally and five intravenously, but both methods are consistent with the single regression line in Fig. 4.

Tanaka et al. [17] used by far the highest dose of vitamin C, 90 g/day. Figure 4 indicates that the substantial benefit observed in that trial may be explained by the particularly sick patients with burns requiring very long ventilation, rather than by the very high vitamin C dose. We found that vitamin C shortened the length of ventilation on average by 25% (*P* = 10^−10^) when the Tanaka trial was excluded and the analysis was restricted to the five trials with dosage from 1 to 6 g/day for patients ventilated for over 10 h [11, 13–15, 18].

The Sadeghpour trial [19] is not included in our statistical models because of the high dropout rate, but the results are shown in Fig. 4 by the dashed line and they are consistent with the findings of the other trials.

### Secondary analysis of vitamin C and E combination trials

**Fig. 5:**
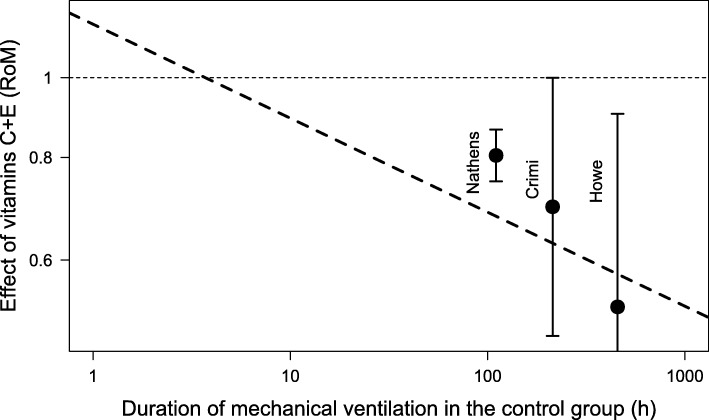
Effect of the combination of vitamins C and E on the duration of ventilation by the duration of ventilation in the control group. The diagonal line shows the meta-regression line based on the vitamin C alone trials from Fig. 4. The horizontal dashed line indicates the null effect. The results of the three trials [29–31] and their 95% CIs are shown. For calculations, see Additional file 2. *RoM* ratio of means [22]

Our meta-regression analysis above was restricted to studies in which vitamin C was administered as the only difference between the study groups. We did not carry out a systematic search for trials on the combination of vitamins C and E, but in our search for the vitamin C trials, we found three trials that administered vitamins C and E together to critically ill patients [29–31]. Given that our search for vitamin C also identifies any combinations of vitamins C and E, it is unlikely that there are many more combination trials. As a secondary analysis, we compared the findings of the three vitamin C and E trials with our meta-regression model based on the eight trials using vitamin C alone (Fig. 5).

Nathens et al. administered 1 g/day vitamin C intravenously and 1000 IU/day vitamin E enterally [29]. The duration of mechanical ventilation in the control group was 110 h, for which our model predicts a 32% decrease in the vitamin C group. The observed decrease in the vitamins C and E group was 20% (95% CI 13% to 26%).

Crimi et al. administered 0.5 g/day vitamin C and 400 IU/day vitamin E enterally [30]. Ventilation time in the control group was 213 h, for which our model predicts a 37% decrease in the vitamin C group. The observed decrease was 30% (95% CI 0% to 61%).

Howe et al. administered 1 g/day vitamin C and 1000 IU/day vitamin E enterally [31]. Ventilation time in the control group was 456 h, and our model predicts a 42% decrease in the vitamin C group. The observed decrease was 47% (95% CI 7% to 87%). In all three vitamin C and E trials, the observed effect of treatment was quite close to the effect predicted by the trials using only vitamin C (Fig. 5; see Additional files 1 and 2 for the calculations).

## Discussion

There is significant variation in the severity of disease in patients who are mechanically ventilated. One measure of severity is the mechanical ventilation time required by the patient, which we used as a proxy for severity. In this study, we found that the duration of ventilation in the untreated control group explained most of the variation in the reported effects of vitamin C on the mechanical ventilation time. In the standard meta-analysis, there is high-level heterogeneity with *I*^2^ = 83% (Fig. 3), whereas in the meta-regression of the vitamin C effect by the control group duration of ventilation, the residual heterogeneity is small with *I*^2^ = 12% (Fig. 4).

Some of the included trials examined elective surgical patients. These patients are not usually critically ill; however, as a result of their surgery, they are routinely ventilated in the ICU for a period of time. In the meta-regression, such patients are located on the left-hand side of Fig. 4 which means that the analysis takes into account the low level of illness severity. In contrast, the inclusion of patients with less severe disease in the standard meta-analysis decreases the average effect of vitamin C, so that the greater effect on the sicker patients is masked (Fig. 3).

The substantial benefit observed in the Tanaka [17] trial seems to be explained by the particularly long mechanical ventilation in the untreated control patients (which reflects the greater illness severity), rather than the particularly high vitamin C dosage of 90 g/day in that trial. All the other trials used 6 g/day or less, but there is no evidence that the benefit was less than in the Tanaka trial when taking into account the ventilation time in the untreated control group (Fig. 4). There are a few reports of deaths caused by intravenous vitamin C in doses of 80 to 224 g/day [32, 33]. Therefore, the interpretation that the benefit in the Tanaka trial may be caused by the type of patients and not by the very high vitamin C dose is important for planning further trials.

Our previous analysis of the length of ICU stay also found that the effect of vitamin C appeared greater for the sicker patients. The length of ICU stay was reduced by 10.1% (*P* = 0.0001) in patients who required an ICU stay of 3 days or longer, but by just 5.7% (*P* = 0.03) in those who needed only 1–2 days in the ICU [1].

There are also other findings that are consistent with vitamin C having a greater effect on patients with more severe medical conditions. A meta-analysis of vitamin C effect on exercise-induced bronchoconstriction found that vitamin C halved FEV_1_ decline caused by exercise [34]. The constant relative effect indicates that the absolute effect was greatest for patients who had the greatest bronchoconstriction in the exercise test. Finally, a trial with common cold patients indicated that the bronchodilatory effect of vitamin C was most beneficial for those with the greatest bronchial hypersensitivity to histamine [35, 36].

There is much evidence indicating that vitamins C and E have an interaction in vitro and in vivo [37–41], and three trials have examined the effect of the combination of vitamins C and E on the duration of mechanical ventilation [29–31]. The reported effects from the three trials are largely consistent with the meta-regression model based on the eight trials using vitamin C alone (Fig. 5), though the confidence interval of the Nathens et al. trial does not cross the regression line. Thus, the statistically significant benefit observed in each of these three trials might be explained by the long ventilation time in the control groups, indicating greater severity of illness in the patients, rather than by the addition of vitamin E to the intervention. To test the possible additional benefit of vitamin E over vitamin C would require 2 × 2 factorial trials.

Although our meta-regression analysis by the ventilation time in the control group explains the heterogeneity in the published trials, it seems evident that other variables influence the effects of vitamin C. For example, there are indications that treatment effects can differ between less and more developed countries. Panagiotou et al. identified several studies that reported greater treatment effects in less developed countries than in more developed countries [42]. Although methodological variations may explain some of the differences, there can also be genuine treatment differences between substantially different cultures, since wealth is strongly correlated with life-style factors including nutrition and with differences in hospital treatments. Previously, vitamin C was found to prevent post-operative atrial fibrillation in non-US trials, but not in US-based trials [43], which may also indicate that the effects of vitamin C can depend on cultural context. Thus, although the fit of the meta-regression line in Fig. 4 is good, the findings should not be extrapolated directly to other contexts.

Two recent meta-analyses concluded that vitamin C is not beneficial for critically ill patients [44, 45], whereas a third concluded that vitamin C was beneficial for sepsis patients [46]. However, all three meta-analyses included studies that administered vitamin C in combination with numerous other substances, such as vitamins A, B, and E, selenium, and zinc [47–49]. Such trials do not test the specific effect of vitamin C. The other substances can have negative or positive effects, and they can also modify the effect of vitamin C. The three meta-analyses also had statistical shortcomings [47–49]. Our current meta-analysis was restricted to trials that tested vitamin C alone. A fourth recent meta-analysis concluded that vitamin C shortens ventilation time in cardiac surgery patients [50]; however, the study was shown to contain several substantial statistical errors [51].

In systematic reviews, one potential concern is publication bias, in that negative trials may remain unpublished. However, publication bias cannot realistically generate the close association shown in Fig. 4. To explain this association by publication bias would require that positive studies with less ill patients remain unpublished, and negative studies with severely ill patients also remain unpublished. Five trials did not use an explicit placebo [12–15, 17], but we do not consider that the lack of placebo undermines the validity of those trials, since ICU patients receive numerous treatments and it is unlikely that one additional tablet or infusion would cause a substantial placebo effect for ventilated patients. The lack of a placebo may cause bias in research on subjective outcomes, but less so on objective outcomes [52]. Thus, it is unlikely to bias studies with outcomes such as the duration of mechanical ventilation.

## Conclusions

It may not be worthwhile to carry out further research on the effects of vitamin C on mechanical ventilation for patient groups that require on average less than 10 h of ventilation. The level of sickness severity should be taken into account in future studies, for example, by evaluating prognostic scores at the start of the trial. Our analysis did not find differences between oral and intravenous vitamin C, but oral administration is rarely an option for the sickest patients, for whom the effects of vitamin C appear greatest. Our analysis is not informative about the optimal dosage of vitamin C. Future trials should directly compare different dosage levels.

## Supplementary information

**Additional file 1 **Calculations; **Table S1.** Detailed description of the included trials.

**Additional file 2.** The data set used in this analysis.

## Data Availability

Descriptions of the included trials, and the risk of bias assessment, and the analyzed data are available as Additional files 1 and 2.
