# Supplementary material for: Vitamin C may reduce the duration of mechanical ventilation in critically ill patients: a meta-regression analysis
Source: J Intensive Care. 2020 Feb 7;8:15. doi: 10.1186/s40560-020-0432-y (PMC7006137; doi:10.1186/s40560-020-0432-y)
Supplement: Supplementary file 1 — Additional file 1 Calculations; Table S1. Detailed description of the included trials. [file 40560_2020_432_MOESM1_ESM.pdf]

Vitamin C may reduce the duration of mechanical ventilation in critically ill patients: a meta-regression analysis

Harri Hemilä [1)] and Elizabeth Chalker [2)]

1. Department of Public Health, University of Helsinki, POB 41,
2. University of Sydney, Sydney, Australia

Submitted to
Journal of Intensive Care
https://jintensivecare.biomedcentral.com/

Harri Hemilä, MD, PhD
Department of Public Health,
University of Helsinki, POB 41,
Helsinki, FI-00014, FINLAND.
E-mail: harri.hemila@helsinki.fi

ver 2020-1-5

Contents **Page**

Standard meta-analysis of 8 trials	2
Calculation of meta-regression, all 8 trials	3
Calculation of meta-regression, 6 trials	4
Calculation of the meta-analysis from 5 trials	5
Table S1: detailed description of the included trials	6

Standard meta-analysis of 8 trials:

```
> venMA <- metagen(TE, seTE, studlab,data=Ven,sm="RR", comb.random=F)
> venMA
             RR             95%-CI %W(fixed)
Amini    1.0973 [0.8572; 1.4047]        6.7
Bjordahl 0.8571 [0.7444; 0.9868]       20.7
Dehghani 0.8683 [0.6685; 1.1278]        6.0
Ebade    1.0251 [0.9261; 1.1346]       39.9
Habib    0.5845 [0.4960; 0.6888]       15.2
Safaei   0.6594 [0.4646; 0.9360]        3.4
Tanaka   0.5681 [0.3549; 0.9095]        1.9
Zabet    0.7830 [0.6057; 1.0122]        6.2

Number of studies combined: k = 8

                        RR             95%-CI     z  p-value
Fixed effect model  0.8644 [0.8107; 0.9216] -4.46 < 0.0001

Quantifying heterogeneity:
tau^2 = 0.0494; H = 2.45 [1.79; 3.36]; I^2 = 83.4% [68.8%; 91.2%]

Test of heterogeneity:
     Q d.f.  p-value
 42.16    7 < 0.0001

Details on meta-analytical method:
- Inverse variance method
>
> (pvalue2sided=2*pnorm(-4.46))  // test for overall effect
[1] 8.2e-06
>
>
> ((1-0.8644)*100)               // estimate and 95% CI in percentages
[1] 13.6
> ((1-0.8107)*100)
[1] 18.9
> ((1-0.9216)*100)
[1] 7.84

> (pchisq(42.16, df=7, lower.tail=FALSE))   // test for heterogeneity
[1] 4.8e-07
```

Calculation of meta-regression, all 8 trials
Regression over log(mechanical ventilation time)

All 8 trials (the Sadeghpour trial is excluded in this analysis)

```
> MetaReg <- metareg(venMA,LogVenti)
> MetaReg

Mixed-Effects Model (k = 8; tau^2 estimator: DL)

tau^2 (estimated amount of residual heterogeneity):     0.0017 (SE = 0.0083)
tau (square root of estimated tau^2 value):             0.0414
I^2 (residual heterogeneity / unaccounted variability): 11.97%
H^2 (unaccounted variability / sampling variability):   1.14
R^2 (amount of heterogeneity accounted for):            96.54%

Test for Residual Heterogeneity:
QE(df = 6) = 6.8156, p-val = 0.3382

Test of Moderators (coefficient(s) 2):
QM(df = 1) = 27.1458, p-val < .0001

Model Results:

          estimate      se     zval    pval    ci.lb    ci.ub
intrcpt     0.1499  0.0714   2.0976  0.0359   0.0098   0.2899    *
LogVenti   -0.2633  0.0505  -5.2102  <.0001  -0.3623  -0.1642  ***

---
Signif. codes:  0 '***' 0.001 '**' 0.01 '*' 0.05 '.' 0.1 ' ' 1

>
> options(digits=6)
> (pvalue2sided=2*pnorm(-5.2102)) #test for assocition with ventilation
[1] 1.88e-07
```

Calculation of meta-regression, 6 trials
Regression over log(mechanical ventilation time)
The trials by Habib [5] and Tanaka [10] with >100 hour ventilation are excluded

```
> Ven100 <- subset(Ven, Ventilation <100)
> venMA100 <- metagen(TE, seTE, studlab,data=Ven100,sm="RR", comb.random=F)
> venMA100
             RR           95%-CI %W(fixed)
Amini    1.0973 [0.8572; 1.4047]       8.1
Bjordahl 0.8571 [0.7444; 0.9868]      25.0
Dehghani 0.8683 [0.6685; 1.1278]       7.3
Ebade    1.0251 [0.9261; 1.1346]      48.1
Safaei   0.6594 [0.4646; 0.9360]       4.0
Zabet    0.7830 [0.6057; 1.0122]       7.5

Number of studies combined: k = 6

                        RR           95%-CI     z p-value
Fixed effect model 0.9375 [0.8738; 1.0059] -1.80  0.0726

Quantifying heterogeneity:
tau^2 = 0.0135; H = 1.56 [1.00; 2.45]; I^2 = 59.0% [0.0%; 83.3%]

Test of heterogeneity:
     Q d.f. p-value
 12.19    5  0.0323

Details on meta-analytical method:
- Inverse variance method
> MetaReg100 <- metareg(venMA100,LogVenti)
> MetaReg100

Mixed-Effects Model (k = 6; tau^2 estimator: DL)

tau^2 (estimated amount of residual heterogeneity):     0 (SE = 0.0099)
tau (square root of estimated tau^2 value):             0
I^2 (residual heterogeneity / unaccounted variability): 0.00%
H^2 (unaccounted variability / sampling variability):   1.00
R^2 (amount of heterogeneity accounted for):            100.00%

Test for Residual Heterogeneity:
QE(df = 4) = 3.8995, p-val = 0.4198

Test of Moderators (coefficient(s) 2):
QM(df = 1) = 8.2875, p-val = 0.0040

Model Results:

          estimate      se     zval    pval    ci.lb    ci.ub
intrcpt     0.0902  0.0646   1.3951  0.1630  -0.0365   0.2168
LogVenti   -0.1801  0.0626  -2.8788  0.0040  -0.3028  -0.0575  **
```

Calculation of the meta-analysis from 5 trials
The trial by Tanaka [10] and trials with <10 hours ventilation are excluded
Thus, this limits to 1 to 6 g/day trials with control group ventilation time >10 hours

```
> Ven10 <- subset(Ven, Ventilation >10&Ventilation <500)
> Ven10
# A tibble: 5 x 12
  studlab  participants   RoM   seTE Ventilation route  dose  long     TE      hi     lo LogVenti
  <chr>    <chr>        <dbl>  <dbl>       <dbl> <chr> <dbl> <dbl>  <dbl>   <dbl>  <dbl>    <dbl>
1 Bjordahl cardiac      0.857 0.0719        33.6 po        2 85710 -0.154 -0.0133 -0.295     1.53
2 Dehghani cardiac      0.868 0.133         15.4 po        1 86830 -0.141  0.120  -0.403     1.19
3 Habib    non-cardiac  0.584 0.0838       189.  iv        6 58450 -0.537 -0.373  -0.701     2.28
4 Safaei   cardiac      0.659 0.179         22.9 iv        2 65940 -0.416 -0.0662 -0.767     1.36
5 Zabet    non-cardiac  0.783 0.131         46.8 iv        6 78300 -0.245  0.0121 -0.501     1.67

> venMA10 <- metagen(TE, seTE, studlab,data=Ven10,sm="RR")

> venMA10
             RR            95%-CI %W(fixed) %W(random)
Bjordahl 0.8571 [0.7444; 0.9868]      40.2       25.1
Dehghani 0.8683 [0.6685; 1.1278]      11.7       18.4
Habib    0.5845 [0.4960; 0.6888]      29.6       23.8
Safaei   0.6594 [0.4646; 0.9360]       6.5       14.1
Zabet    0.7830 [0.6057; 1.0122]      12.1       18.6

Number of studies combined: k = 5

                         RR            95%-CI     z  p-value
Fixed effect model   0.7454 [0.6817; 0.8150] -6.45 < 0.0001
Random effects model 0.7432 [0.6195; 0.8916] -3.20   0.0014

Quantifying heterogeneity:
tau^2 = 0.0292; H = 1.88 [1.18; 2.99]; I^2 = 71.7% [28.4%; 88.8%]

Test of heterogeneity:
     Q d.f. p-value
 14.11    4  0.0069

Details on meta-analytical method:
- Inverse variance method
- DerSimonian-Laird estimator for tau^2
```

Table S1: detailed description of the included trials

Amini 2018

**Methods**	Randomized trial with 4-arms: vitamin C, NAC, selenium and control; we restricted our analysis to vitamin C and control arms<br>https://doi.org/10.21470/1678-9741-2017-0071<br>https://www.ncbi.nlm.nih.gov/pmc/articles/PMC5985838<br>https://www.ncbi.nlm.nih.gov/pubmed/29898141
**Participants**	Iran, CABG patients, 56 M / 51 F; mean age: 60 yr (SD 10 yr); 67 vit C / 70 control. One control male (#198) was removed, see Notes<br><br>**Inclusion:** NYHA class of I-III undergoing elective off-pump CABG. Patients underwent standard intravenous anesthesia and were transferred to ICU for post-cardiac surgery for further monitoring and recovery.<br><br>**Exclusion:** Change from off-pump to on-pump surgery, known drug allergy, history of COPD, anemia, congestive heart failure (CHF), active sepsis, preoperative ejection fraction lower than 40%, preoperative creatinine above 1.3 mg/dL, use of nephrotoxic drugs, coronary angiography, intraoperative transfusion of more than 2 units of red blood cells, perioperative use of intra-aortic balloon counterpulsation, perioperative requirement for high-dose vasopressors, and any intraoperative life-threatening events such as fatal arrhythmias, excessive bleeding, or desaturation.
**Interventions**	**Vit C before the operation:**<br>**Dose:** 3 g/d "vitamin C... 1500 mg ... tablets, twice a day, from 24 hours before the operation until two postoperative days."<br>**Method:** po<br>**Timing:** "24 h before operation"<br>**Vit C after operation:**<br>**Dose:** 3 g/d "1500 mg .. tablets ... twice a day"<br>**Method:** po<br>**Duration:** 2 d "until two postoperative days"<br>**Control group:** no tablets. The patients received many drugs and it is unlikely that they identified vitamin C among all the other administered drugs. We classify that all the other drugs serve as a functional placebo to vitamin C.
**Outcomes**	Length of mechanical ventilation

**Notes**	"Ventilation times were 7.33±6.02, 10.68±27.15, 5.90±3.13, and 25.36±157.5 hours in the vitamin C, NAC, selenium and control groups, respectively (P=0.429)." (p 131)<br><br>Thus, the SD for ventilation in the control group was much greater than the SD for each of the other 3 groups.<br><br>We were able to contact Dr. Amini and he kindly sent us their data set (email 2018-11-6). We found that there was one patient (#198 male) in the control group who had ventilation time 1333 hours. We removed this outlier and calculated that mean ventilation time 6.68 (SD 4.26) hours in the control group. This revised SD values is closely similar to the SD values of the three other arms, compare above.<br><br>We were also able to get more information of the Methods from Dr. Amini (email 2018-12-13), see below.

Risk of bias table

Bias	Authors' judgement	Support for judgement
Random sequence generation (selection bias)	Low risk	"Using a computer-based randomization method" (p 130).<br><br>Groups were balanced (Table 2) for eg. age, sex, weight, height, baseline diseases, ejection fraction
Allocation concealment (selection bias)	Low risk	"The patients, the researcher who collected the data and the one who analyzed the data were blinded to the study. However, the physicians and nurses in charge were aware of the allocated groups" (email 2018-12-13).
Blinding of participants and personnel (performance bias)	Low risk	"The patients, the researcher who collected the data and the one who analyzed the data were blinded to the study. However, the physicians and nurses in charge were aware of the allocated groups" (email 2018-12-13).<br><br>Given that no benefit was found from vitamin C, we do not consider that this lack of benefit could be biased by "the physicians and nurses in charge were aware of the allocated groups". Therefore we classify this as low risk for personnel.
Blinding of outcome assessment (detection bias)	Low risk	"The patients, the researcher who collected the data and the one who analyzed the data were blinded to the study. However, the physicians and nurses in charge were aware of the allocated groups" (email 2018-12-13).<br><br>Given that no benefit was found from vitamin C, we do not consider that this lack of benefit could be biased by "the physicians and nurses in charge were aware of the allocated groups". Therefore we classify this as low risk for outcome assessment.
Incomplete outcome data (attrition bias)	Low risk	6 vitamin C participants and 2 control participants discontinued (p 131; Fig 1) and we (HH and EC) removed 1 control participant, see Notes above.

Bjordahl 2012

**Methods**	Triple-blind, randomised placebo-controlled trial, October 2009 to April 2011.<br>http://dx.doi.org/10.1016/j.amjsurg.2012.03.012<br>http://www.ncbi.nlm.nih.gov/pubmed/23022248
**Participants**	USA, CABG patients, 124 M / 61 F; mean 63 yr (SD 12 yr); 89 vit C / 96 placebo.<br><br>**Inclusion:** >18 yr who were scheduled to undergo CABG.<br><br>**Exclusion:** current AF, temporary or permanent pacemaker, life expectancy <1 month, emergency surgery precluding the initiation of study protocol the evening before surgery, current pregnancy.
**Interventions**	**Vit C before the operation:**<br>**Dose:** 2 g<br>**Method:** po<br>**Timing:** "the evening before surgery"<br>**Vit C after operation:**<br>**Dose:** 2 g/d "1 gram twice daily"<br>**Method:** po<br>**Duration:** 5 d<br>**Placebo:** "identical placebo capsules at the same intervals"; "the inert substance for both treatment and placebo capsules was talc."
**Outcomes**	Length of mechanical ventilation
**Notes**	"Both ascorbic acid and inert placebo capsules were prepared by a custom pharmacy" (p 863) indicates that the products were not commercial.<br>Bjorndahl reported the duration of ventilation in vitamin C group as 1.2 (SD 0.8) d and in control group as 1.4 (SD 1.0) d.<br>Calculation of the P-value with the Taylor series formula from these reported mean and SD-values gives P = 0.13.<br>Bjorndahl reported P = 0.032 (Mann Whitney) for the effect of vitamin C on ICU stay.<br>We used this P = 0.032 to calculate the consistent SE(log(RoM), see Supplementary file.

Risk of bias table

Bias	Authors' judgement	Support for judgement
Random sequence generation (selection bias)	Low risk	"Enrolled participants were randomized to either ... The pharmacy department maintained the randomization list ..." (p 863).<br><br>Groups were balanced (Table 1) for eg. age, sex, BMI, baseline diseases, ejection fraction
Allocation concealment (selection bias)	Low risk	"The pharmacy department maintained the randomization list and assigned participants to the placebo and treatment arms of the study in a blinded fashion. Participants, clinicians, and evaluators were blinded to the treatment assignments and the blind was not broken until after data analyses were complete" (p 863).
Blinding of participants and personnel (performance bias)	Low risk	See above.
Blinding of outcome assessment (detection bias)	Low risk	See above.
Incomplete outcome data (attrition bias)	Low risk	13 participants were withdrawn from analysis because of surgery postponement/cancellation (3), presence of exclusion criteria at the time of enrollment (4), protocol violation (1), cardiac surgery without CABG (3), use of bloodless therapy for religious purposes (1) and intraoperative exsanguination and death unrelated to current study (1).

Dehghani 2014

**Methods**	Randomised trial, March 2012 to March 2013<br>http://www.ncbi.nlm.nih.gov/pubmed/24293167<br>http://dx.doi.org/10.5603/CJ.a2013.0154<br>http://czasopisma.viamedica.pl/cj/article/view/36075
**Participants**	Iran, CABG patients, 74 M / 26 F, mean age: 61 yr (SD 7 yr); 50 vit C / 50 control.<br><br>**Inclusion:** Patients who underwent elective isolated on-pump CABG surgery, age >50 yr, no history of CABG surgery, taking beta-blocker before and after surgery.<br><br>**Exclusion:** history of any cardiac arrhythmia and/or being under anti-arrhythmic therapy, being under digoxin therapy, having pacemaker, severe CHF and/or LVEF <30%, renal failure, severe hepatic failure, COPD, no occurrence of intra- or post-operative cardiopulmonary arrest, or any degree of cardiac blockade and/or bradycardia.
**Interventions**	**Vit C before the operation:**<br>**Dose:** 2 g "2 g of vitamin C tablets before the surgery"<br>**Method:** po<br>**Timing:** "All patients took the tablets within 12 hours before surgery" (email 2015-9-9)<br>**Vit C after operation:**<br>**Dose:** 1 g/d "500 mg twice daily"<br>**Method:** po<br>**Duration:** 5 d<br>**Placebo:** No tablets. The patients received many drugs and it is unlikely that they identified vitamin C among all the other administered drugs. We classify that all the other drugs serve as a functional placebo to vitamin C.
**Outcomes**	Length of mechanical ventilation
**Notes**	"Our study was funded by Urmia University of Medical Sciences, Iran" (email 2015-10-1).<br><br>Additional information was received by emails from Yousef Rezaei (2015-4-11, 2015-4-22 and 2015-9-9), see above and below.

Risk of bias table

Bias	Authors' judgement	Support for judgement
Random sequence generation (selection bias)	Low risk	"All patients were randomized into two groups in a 1:1 ratio using random-number table" (p 493).<br><br>Groups were balanced (Table 1) for eg. age, sex, smoking, baseline diseases, ejection fraction
Allocation concealment (selection bias)	Low risk	"Neither ward physician nor Holter interpreter were aware of the patients' group. Only one who analyzed data was aware of the patients' group" (email 2015-4-11) and<br><br>"we did not let ward physician and surgeons to know which of patients taking vitamin c or not, except for being informed about the conduction of our trial and prescribing some of patients to take vitamin c. Furthermore, patients were informed that they would be included in our trial to be prescribed vitamin c" (email 2015-4-22).
Blinding of participants and personnel (performance bias)	Low risk	"Neither ward physician ... were aware of the patients' group. " (email 2015-4-11) and<br><br>"we did not let ward physician and surgeons to know which of patients taking vitamin c or not, except for being informed about the conduction of our trial and prescribing some of patients to take vitamin c. Furthermore, patients were informed that they would be included in our trial to be prescribed vitamin c" (email 2015-4-22).
Blinding of outcome assessment (detection bias)	Low risk	See above.
Incomplete outcome data (attrition bias)	Low risk	"There was no patient withdrawal or missing during study. All allocated ones completed study" (email 2015-4-11 and 2015-10-1).

Ebade 2014

**Methods**	Controlled trial, July 2010 to December 2013<br>http://www.ejca.eg.net/article.asp?issn=1687-9090;year=2014;volume=8;issue=2;spage=59;epage=65;aulast=Ebade
**Participants**	Egypt, CABG patients, 29 M / 11 F; mean age: 55 yr (SD 10 yr); 20 vit C / 20 control.<br><br>**Inclusion:** patients assigned for elective CABG surgery with cardiopulmonary bypass (CPB) were enrolled in the study.<br><br>**Exclusion:** renal or hepatic dysfunction, known hypersensitivity to the studied drugs, COPD, preoperative AF, pacemaker, class I and III antiarrhythmic agents or digoxin, any degree of AV block
**Interventions**	**Vit C before the operation:**<br>**Dose:** 2 g<br>**Method:** iv, but not explicitly stated; "after induction of anesthesia" implies iv administration<br>**Timing:** "after induction of anesthesia"<br>**Vit C after operation:**<br>**Dose:** 3 g/d "1 g every 8 h daily until the fifth post-operative day" (p 60).<br>**Method:** iv, but not explicitly stated. "Control group receiving saline infusion" suggests that vitamin C also was administered by infusion<br>**Duration:** 5 d<br>**Placebo** saline infusion
**Outcomes**	Length of mechanical ventilation
**Notes**	Very poorly reported study.<br><br>We tried to contact Dr. Ebade to ask for the details of their methods (2015-10-30, 2015-11-1, 2015-11-18, 2015-11-23 and 2019-1-9), but we did not get any response.

Risk of bias table

Bias	Authors' judgement	Support for judgement
Random sequence generation (selection bias)	Unclear risk	"divided into three equal groups" (p 60) (One group received magnesium). However, Ebade did not describe the method of allocation; alternative allocation?<br><br>Groups were balanced (Table 2) for eg. age, sex, weight, height, ejection fraction.<br><br>Because of the poor reporting, we classify as unclear.
Allocation concealment (selection bias)	Unclear risk	Not described
Blinding of participants and personnel (performance bias)	Unclear risk	Not described
Blinding of outcome assessment (detection bias)	Unclear risk	Not described
Incomplete outcome data (attrition bias)	Unclear risk	No description whether the 40 reported patients equals the number of randomized participants, no flow diagram

Habib 2017

**Methods**	Quasi-randomized trial<br>http://dx.doi.org/10.19070/2329-9967-1700015
**Participants**	Egypt, sepsis patients, 58 M / 42 F; mean age 42 y (SD 10 y); 50 vit C / 50 control<br><br>**Inclusion:** patients admitted to the critical care department with the diagnosis of septic shock<br><br>Exclusions: Pregnant and lactating mothers, patients with a history of oxalate nephrolithiasis or in documented glucose-6-phosphate dehydrogenase (G6PD) deficiency, paroxysmal nocturnal hemoglobinuria, and hereditary hemochromatosis. Patients with other types of shock state or patients with mixed shock.
**Interventions**	**Vit C dosage:**<br>**Dose:** "1.5 gm vitamin C"<br>**Method:** iv<br>**Timing:** "every 6 hours" (p 78) Thus, total vitamin C daily dose was 6 g/day.<br>**Placebo** no explicit placebo. "Control group: 50 septic shock patients received conventional sepsis treatment only" (p 78).<br>The patients received many drugs and it is unlikely that they identified vitamin C among all the other administered drugs. We classify that all the other drugs serve as a functional placebo to vitamin C.
**Outcomes**	Length of mechanical ventilation
**Notes**	Quite poorly reported study.<br><br>We tried to contact Dr. Habib to ask for the details of their methods (2019-3-29 and 2019-4-1 and 2019-4-10), but we did not get any response.

Risk of bias table

Bias	Authors' judgement	Support for judgement
Random sequence generation (selection bias)	Low risk	Quasi-randomization: "All enrolled patients who met the inclusion criteria (n=100) were randomized (even odd randomization technique) into the study" (p 78).<br><br>Groups were balanced (Table 1) for age, sex, APACHE II score, SOFA score, mean arterial pressure, heart rate, respiratory rate, temperature, urine output, (Table 2) CRP and procalcitonin
Allocation concealment (selection bias)	Unclear risk	Not described
Blinding of participants and personnel (performance bias)	Unclear risk	Not described
Blinding of outcome assessment (detection bias)	Unclear risk	Not described
Incomplete outcome data (attrition bias)	Low risk	The report indicates that 100 participants were enrolled "All enrolled patients who met the inclusion criteria (n=100)", and there are 100 participants in their analysis

Sadeghpour 2015 (not included in the statistical models for the 42% dropout rate)

**Methods**	Double-blind randomised placebo-controlled trial.<br>https://doi.org/10.5812/aapm.25337<br>http://www.ncbi.nlm.nih.gov/pmc/articles/PMC4350190<br>http://www.ncbi.nlm.nih.gov/pubmed/25789244<br>Duplicate publication in Farsi (Persian):<br>Moludi J , Keshavarz S , Pakzad R , Sedghi N , Sadeghi T and Alimoradi F. Effect of vitamin C supplementation in the prevention of atrial fibrillation [in Persian] Tehran Univ Med J, 2016;73(11):791<br>http://tumj.tums.ac.ir/browse.php?a_code=A-10-25-5413&sid=1&slc_lang=en
**Participants**	Iran, CABG or valvular surgery patients, 191 M / 99 F, mean age: vitamin c 57 yr (SD 14 yr), placebo 54 yr (SD 14yr); 113 vit C / 177 placebo.<br><br>**Inclusion:** >18 yr with American Society of Anesthesiologists physical status class II-III and candidacy for CABG or simple congenital valvular disease surgery.<br><br>**Exclusion:** who died within the 1st postoperative day and those who had not received adequate doses of drugs according to our protocol, severe complications (cardiac, respiratory or neurological) or emergency operation.
**Interventions**	**Vit C before the operation:**<br>**Dose:** 2 g<br>**Method:** iv<br>**Timing:** "immediately before surgery"<br>**Vit C after operation:**<br>**Dose:** 1 g/d<br>**Method:** po<br>**Duration:** 4 d<br><br>**Placebo:** "The patients in the placebo group received an equal number of identical tablets. The placebo tablets and ampoules were prepared in the same shape and size as the original" (p 2).
**Outcomes**	Length of mechanical ventilation
**Notes**	"We paid it by ourselves besides getting help from the Rajaei cardiovascular research center." (email 2015-10-1)<br>Additional information was received by email from Anita Sadeghpour (2015-5-12 and 2015-10-1), see below and above.<br>In 2019 we received a response describing that 42% of participants were excluded after randomization. The publication describes 113 vit C / 177 placebo participants which indicates that there is substantial difference in the exclusion rate between the vitamin C and placebo groups.<br>This is such a severe violation of the ITT principle that we exclude the trial from our calculations, but we show the results in Fig. 1.

Risk of bias table

Bias	Authors' judgement	Support for judgement
Random sequence generation (selection bias)	Low risk	"The study population was randomized one day before surgery to two groups (By using www.randomizer.org) ... The method of randomization was balanced block with an allocation sequence based on a block size of eight" (p 2).<br>However, the sizes of the groups 113 vit C / 177 placebo are not consistent with block randomization. Therefore, we exclude this study in our sensitivity analysis.<br>Groups were balanced (Table 2) for sex, age, hypertension, diabetes, hyperlipidemia, surgery type were balanced
Allocation concealment (selection bias)	Low risk	"Both the patients and the hospital staff were blind to the treatment allocation" (p 2).
Blinding of participants and personnel (performance bias)	Low risk	"Both the patients and the hospital staff were blind to the treatment allocation" (p 2).<br>"The Vit C was given in the operating room along with the other infusions by anesthesiologist technician" (email 2015-5-12).<br>"The patients in the intervention group received 2 g of vitamin C ... intravenously, immediately before surgery in the operating theatre, followed by 1 g daily oral doses of the tablets for the first four postoperative days. The patients in the placebo group received an equal number of identical tablets. The placebo tablets and ampoules were prepared in the same shape and size as the original ones manufactured by the same pharmaceutical company." (p 2).
Blinding of outcome assessment (detection bias)	Low risk	See above.
Incomplete outcome data (attrition bias)	High risk	"we enrolled 500 patients but we excluded the patients who died on the first postoperative day, those who needed re operation due to technical problems and excessive bleeding, and those who had not received an adequate dose." (email 2019-1-24).<br>Thus, data is published of 290 participants of 500 enrolled, which means that data of 42% of participants are missing.<br>This is such a severe violation of the ITT principle that we exclude the trial from our nalysis.

Tanaka 2000

**Methods**	Quasi-randomized trial, Dec 1992 to Dec 1997<br>https://doi.org/10.1001/archsurg.135.3.326<br>https://www.ncbi.nlm.nih.gov/pubmed/10722036
**Participants**	Japan, patients with burns, 25 M / 12 F; mean age: 45 yr (SD 22 yr); 19 vit C / 18 control.<br><br>**Inclusion:** "older than 16 years; thermal injury within 2 hours before admission; burn covering greater than 30% of total body surface area"<br><br>**Exclusion: "**preexisting hepatic, respiratory, cardiac, or renal dysfunction; and preexisting coagulopathy"
**Interventions**	**Vit C dosage:**<br>**Dose:** "66 mg/kg per hour" corresponds to 110 g/day for a 70 kg patient<br>**Method:** iv<br>**Timing:** "24-hour study period" (p 327). Thus, vitamin C was administered for just 1 day.<br>**Placebo** no specific placebo, but functional placebo: "The control group did not receive the ascorbic acid infusion", but "The administered volume of ascorbic acid was included in the 24-hour fluid intake calculations" (p 327). This indicates that patients could not distinguish vitamin C administration by difference in volumes
**Outcomes**	Length of mechanical ventilation
**Notes**	

Risk of bias table

Bias	Authors' judgement	Support for judgement
Random sequence generation (selection bias)	Low risk	Quasi-randomization: "Randomization was performed according to the month of admission" (p 327). It is unlikely that randomization of burn patients according to the month of admission causes systematic bias.<br><br>Groups were balanced at baseline (Table 1 and Fig 1) for age, sex, weight, kinds of burns, areas of burns, thickness of burns, the number of inhalation injuries, heart rate, mean arterial pressure
Allocation concealment (selection bias)	Low risk	Quasi-randomization: "Randomization was performed according to the month of admission" (p 327). It is unlikely that randomization of burn patients according to the month of admission causes systematic bias. There were no baseline difference in age, sex, weight, kinds of burns, areas of burns, thickness of burns, or in the rate of inhalation injuries (Table 1 and Fig 1).
Blinding of participants and personnel (performance bias)	Low risk	"The administered volume of ascorbic acid was included in the 24-hour fluid intake calculations" (p 327). This means that patients had same amount of fluids in both groups. In addition, it is highly unlikely that patient with a burn covering greater than 30% TBSA would observe their treatments.
Blinding of outcome assessment (detection bias)	Unclear risk	Not described
Incomplete outcome data (attrition bias)	Unclear risk	No description whether the 37 reported patients equals the number of allocated participants, no flow diagram

Zabet 2016

**Methods**	Double-blind randomized trial, September 2014 to January 2016<br>https://dx.doi.org/10.4103/2279-042X.179569<br>https://www.ncbi.nlm.nih.gov/pmc/articles/PMC4843590<br>https://www.ncbi.nlm.nih.gov/pubmed/27162802
**Participants**	Iran, Sepsis patients, 21 M / 7 F; mean age: 64 yr (SD 16 yr); 14 vit C / 14 control.<br><br>**Inclusion:** (18–65-year-old) surgical critically ill patients with diagnosis of septic shock who needed a vasopressor drug to maintain mean arterial pressure >65 mmHg despite adequate fluid resuscitation were recruited<br><br>**Exclusion:** Not described
**Interventions**	**Vit C at the ICU:**<br>**Dose:** "25 mg/kg intravenous ascorbic acid every 6 h for 72 hours" corresponds to 7 g/day for a 70 kg patient<br>**Method:** iv<br>**Duration:** 72 hours<br>**Placebo** "Patients in the placebo group received 50 ml of dextrose 5% solution as intravenous infusion"
**Outcomes**	Length of mechanical ventilation
**Notes**	

Risk of bias table

Bias	Authors' judgement	Support for judgement
Random sequence generation (selection bias)	Low risk	"according to the permuted block randomization. The randomization scheme consisted of seven blocks and each block contained four patients in random order" (p 95).<br><br>Groups were balanced (Table 1) for age, sex, APACHE II score, SOFA score, baseline diseases, source of sepsis, time from ICU admission to the diagnosis of sepsis
Allocation concealment (selection bias)	Low risk	"The researchers, ICU nurses, and physicians were blinded regarding the intervention and the product (ascorbic acid or placebo)" (p 95)
Blinding of participants and personnel (performance bias)	Low risk	"The researchers, ICU nurses, and physicians were blinded regarding the intervention and the product (ascorbic acid or placebo)" (p 95)
Blinding of outcome assessment (detection bias)	Low risk	"The researchers, ICU nurses, and physicians were blinded regarding the intervention and the product (ascorbic acid or placebo)" (p 95)
Incomplete outcome data (attrition bias)	Low risk	No drop-outs
